# HspB8 interacts with BAG3 in a “native‐like” conformation forming a complex that displays chaperone‐like activity

**DOI:** 10.1002/pro.4687

**Published:** 2023-07-01

**Authors:** Barbara Sciandrone, Diletta Ami, Annalisa D'Urzo, Elena Angeli, Annalisa Relini, Marco Vanoni, Antonino Natalello, Maria Elena Regonesi

**Affiliations:** ^1^ Department of Biotechnologies and Biosciences University of Milano‐Bicocca Milan Italy; ^2^ Department of Physics University of Genoa Genoa Italy; ^3^ ISBE‐SYSBIO Centre of Systems Biology Milan Italy; ^4^ Milan Center of Neuroscience (NeuroMI) Milan Italy

**Keywords:** autophagy, chaperone‐like activity, HspB8‐BAG3 complex, neurodegenerative diseases, sHsps

## Abstract

The HspB8‐BAG3 complex plays an important role in the protein quality control acting alone or within multi‐components complexes. To clarify the mechanism underlying its activity, in this work we used biochemical and biophysical approaches to study the tendency of both proteins to auto‐assemble and to form the complex. Solubility and Thioflavin T assays, Fourier transform infrared spectroscopy and atomic force microscopy analyses clearly showed the tendency of HspB8 to self‐assemble at high concentration and to form oligomers in a “native‐like” conformation; otherwise, BAG3 aggregates poorly. Noteworthy, also HspB8 and BAG3 associate in a “native‐like” conformation, forming a stable complex. Furthermore, the high difference between dissociation constant values of HspB8‐HspB8 interaction with respect to the binding to BAG3 obtained by surface plasmon resonance confirms that HspB8 is an obligated partner of BAG3 in vivo. Lastly, both proteins alone or in the complex are able to bind and affect the aggregation of the Josephin domain, the structured domain that triggers the ataxin‐3 fibrillation. In particular, the complex displayed higher activity than HspB8 alone. All this considered, we can assert that the two proteins form a stable assembly with chaperone‐like activity that could contribute to the physiological role of the complex in vivo.

## 1 INTRODUCTION

During evolution, cells have developed a series of mechanisms to regulate protein folding, stability and turnover and to prevent the accumulation of toxic aggregates (Dobson, 2003). To date, it is largely accepted that the onset of many neurodegenerative diseases (NDs) is triggered by the inefficiency or failure of cellular protein quality control (PQC): the alteration of proteostasis and the incapacity to re‐establish the physiological condition led to cellular stress and, in most cases, to neuronal death (Balchin et al., 2016; Dobson, 2003). In PQC systems, chaperones cooperate with the degradative pathways, including the ubiquitin‐proteasome system (UPS) and the autophagy. Mutations in chaperones (Smith et al., 2015) and an imbalance of the two degradative pathways play deleterious effects in several NDs (Ciechanover & Kwon, 2015; Senft & Ronai, 2015). In particular, the accumulation of autophagosomes shown in the brains of patients with different NDs, including Alzheimer's disease, transmissible spongiform encephalopathies, Parkinson's disease, and Huntington's disease (Rubinsztein et al., 2007; Williams et al., 2006) strongly underlines the contribution of the autophagy impairment in the pathogenesis of these disorders. On the other hand, the pharmacological activation of autophagy induces a significant decrease of the aggregated species of the proteins associated with several NDs, reducing their cellular toxicity in vitro and their neurotoxicity in animal models (Hochfeld et al., 2013; Rose et al., 2010; Rubinsztein et al., 2007). In this context, the selective macroautophagy plays an important role that includes chaperone‐mediated autophagy (CMA) and two similar processes, endosomal microautophagy (e‐MI) and chaperone‐assisted selective autophagy (CASA). In particular, the CASA complex is directly involved in amyloid aggregates clearance in several NDs (Carra, Seguin, Lambert, & Landry, 2008a; Gamerdinger et al., 2009). The co‐chaperone BAG3 (Bcl‐2 associated athanogene protein 3) is the scaffold of the complex. It belongs to the BAG family, a group of proteins characterized by the presence of protein–protein interaction motifs, separated by long disordered tracts (McCollum et al., 2009). It contains a BAG domain in its C‐terminal region responsible for the binding with the anti‐apoptotic BCL‐2 and the ATPase domain of the HSC/HSP70 chaperone (Doong et al., 2002; Jin et al., 2015; Lee et al., 1999; Takayama et al., 1999). Moreover, the human BAG3 contains three other amino acid motifs and domains: a WW domain which interacts with proteic proline‐rich repeats, a PxxP region which is the binding site of dynein (Gamerdinger et al., 2011) and two conserved IPV motifs that mediate the binding with HspB8 (Fuchs et al., 2009). The last one is an ATP‐independent chaperone, whose expression is induced in response to cellular stress, aging, and in some protein aggregation disorders (Cox et al., 2016). It contains an α‐crystallin domain, formed by 7 β‐strands combined in 2 β‐sheets, flanked by N‐terminal and C‐terminal unordered regions, which are involved in substrate recognition and interaction with other chaperons (Haslbeck et al., 2005; Kazakov et al., 2009). HspB8 belongs to the small heat shock proteins (sHsps) family, small chaperones that function as “holdases,” a term that refers to their capability to prevent protein aggregation by stabilizing partially folded proteins (Cox et al., 2016; Kulig & Ecroyd, 2012). HspB8 exhibits this chaperon‐like activity on several proteins, as already demonstrated in vitro (Chowdary et al., 2004; Kim et al., 2004; Sanbe et al., 2007; Webster et al., 2020), and in vivo (Carra et al., 2005). Furthermore, almost all of the sHsps exist in the form of larger homo‐or hetero‐oligomers containing other sHsps partners. The homo‐oligomers are regarded as reservoir in rapid exchanges with the chaperone‐active dissociated forms (Bova et al., 2000; Bova et al., 2002; Van Montfort et al., 2001). Unlike most Hsps, HspB8 predominantly presents an equilibrium mixture of monomers, dimers, or small aggregates and this seems to be due to the lack of the I/V‐X‐I/V motif in the C‐terminal (Chowdary et al., 2004). Moreover, the evidence that HspB8 formed tight complexes with BAG3 in mammalian cells (Carra, Seguin, Lambert, & Landry, 2008a) supports the model that BAG3 is an obligate partner of HspB8 (Shemetov & Gusev, 2011). According with this, several evidence suggest that BAG3‐HspB8 complex could play different functions in proteostasis, that is, in NDs and in the remodeling of the actin cytoskeleton (Carra et al., 2009; Carra et al., 2010; Carra, Seguin, Lambert, & Landry, 2008a; Carra, Seguin, & Landry, 2008b; Guilbert et al., 2018; Seidel et al., 2012). Nevertheless, little is known about the mode of interaction of the two proteins and of the mechanism of action of the complex. In this work we carried out size exclusion chromatography (SEC), circular dichroism (CD), Fourier transform infrared (FTIR) spectroscopy, surface plasmon resonance (SPR), thioflavin T analyses and solubility assay to deepen the capacity to self‐assemble of HspB8 and elucidate the nature of its interaction with BAG3. Moreover, we employed the Josephin domain of the ataxin‐3, the poly‐Q containing protein responsible for the spinocerebellar ataxia Type 3 (Matos et al., 2011; Orr & Zoghbi, 2007), as an amyloid protein model to study the chaperone‐like activity of HspB8 alone and in the HspB8‐BAG3 complex.

## 2 RESULTS

### 2.1 HspB8 and BAG3 are characterized by intrinsically disordered and folded domains

**FIGURE 1:**
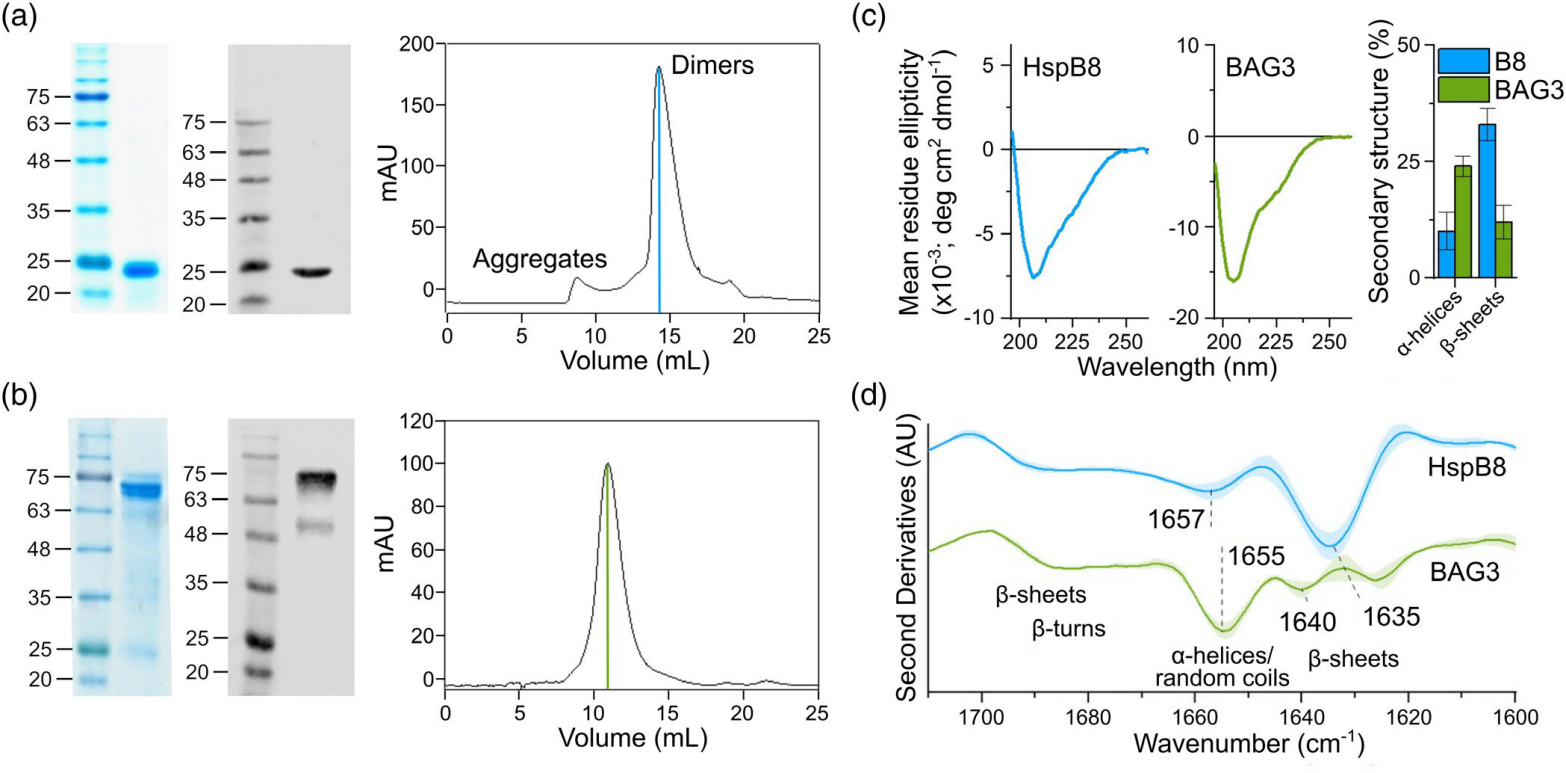
Biochemical and biophysical characterizations of purified HspB8 and BAG3. (a) SDS‐PAGE, western blot, SEC profile of HspB8: 3 μg of the peak obtained by loading samples to column Superose 12 30/100 was subjected to SDS‐PAGE (14%), stained with EZBlue gel staining solution or immunodetected with anti‐HspB8 antibody. Secondary antibody Donkey anti‐Rabbit IRDye at a 1:15,000 dilution was employed. Fluorescence was scanned at 800 nm with Odyssey Fc System (LI‐COR). (b) SDS‐PAGE, western blot. SEC profile of BAG3. 3 μg of the peak obtained from SEC was subjected to SDS‐PAGE (12%) stained with EZBlue gel staining or immunodetected with anti‐BAG3 antibody. (c) Far‐UV CD spectra of HspB8 and BAG3: the percentage of β‐sheet and α‐helical structures obtained by spectral deconvolution is reported in the right panel. Error bars represent the standard deviations from three independent measured spectra. (d) Second derivatives of the FTIR absorption spectra of HspB8 and BAG3 reported in the Amide I band: the wavenumber of the main components and the typical spectral ranges of the different protein secondary structure absorption are reported. The shadowed area refers to the standard deviation from six measured spectra from six independent protein preparations.

HspB8 was cloned in fusion with glutathione S‐transferase whereas BAG3 was obtained as a histidine tagged protein. Size exclusion chromatography (SEC) of HspB8 performed after cleavage with PreScission protease showed a major peak with an apparent molecular weight of 55 kDa that could correspond to the homodimer, and a minor peak found in the empty volume, that could represent the aggregated form of the protein (Figure 1a). SEC profile of BAG3 showed a peak corresponding to an apparent molecular weight of 250 kDa, while the actual value is 74 kDa (Figure 1b). This discrepancy could be ascribed to the highly disordered nature of this protein. The circular dichroism (CD) analysis confirmed the disordered nature of both proteins, whose spectra (Figure 1c) displayed in fact the typical features of disordered conformations (Micsonai et al., 2015; Micsonai et al., 2022). Indeed, disordered proteins exhibit characteristic CD spectra with an intense minimum in the proximity of 200 nm and a low signal around 222 nm. These spectral features are well different from those of typical α‐helical and β‐sheet structures (Ref. Micsonai 2022 Front Mol Biosci 9:863141). However, the quantification of the protein secondary structures by spectral deconvolutions (Micsonai et al., 2015) indicated that HspB8 contains a relatively high amount of β‐sheets (related to the presence of the crystallin domain), while BAG3 is enriched in α‐helical structures (representative of the BAG domain) (Figure 1c). In agreement with these results, both proteins were classified as ordered by the analysis of their far‐UV CD spectra using the disorder–order classification method reported in Micsonai et al. (Micsonai et al., 2022) The ordered structures of the two proteins are clearly detectable in the second derivatives of the Fourier transformed infrared (FTIR) spectra (Ami & Natalello, 2022; Barth, 2007) shown in Figure 1d, where the peak position and assignment to the protein secondary structures of the Amide I band components are also reported. In particular, HspB8 displays a main minimum around 1635 cm^−1^ assigned to intramolecular β‐sheets. BAG3 spectrum, instead, is characterized by a main minimum at around 1655 cm^−1^ mainly assigned to the α‐helical structures of the BAG domain.

### 2.2 HspB8 has a tendency to self‐assemble in “native‐like” conformation

**FIGURE 2:**
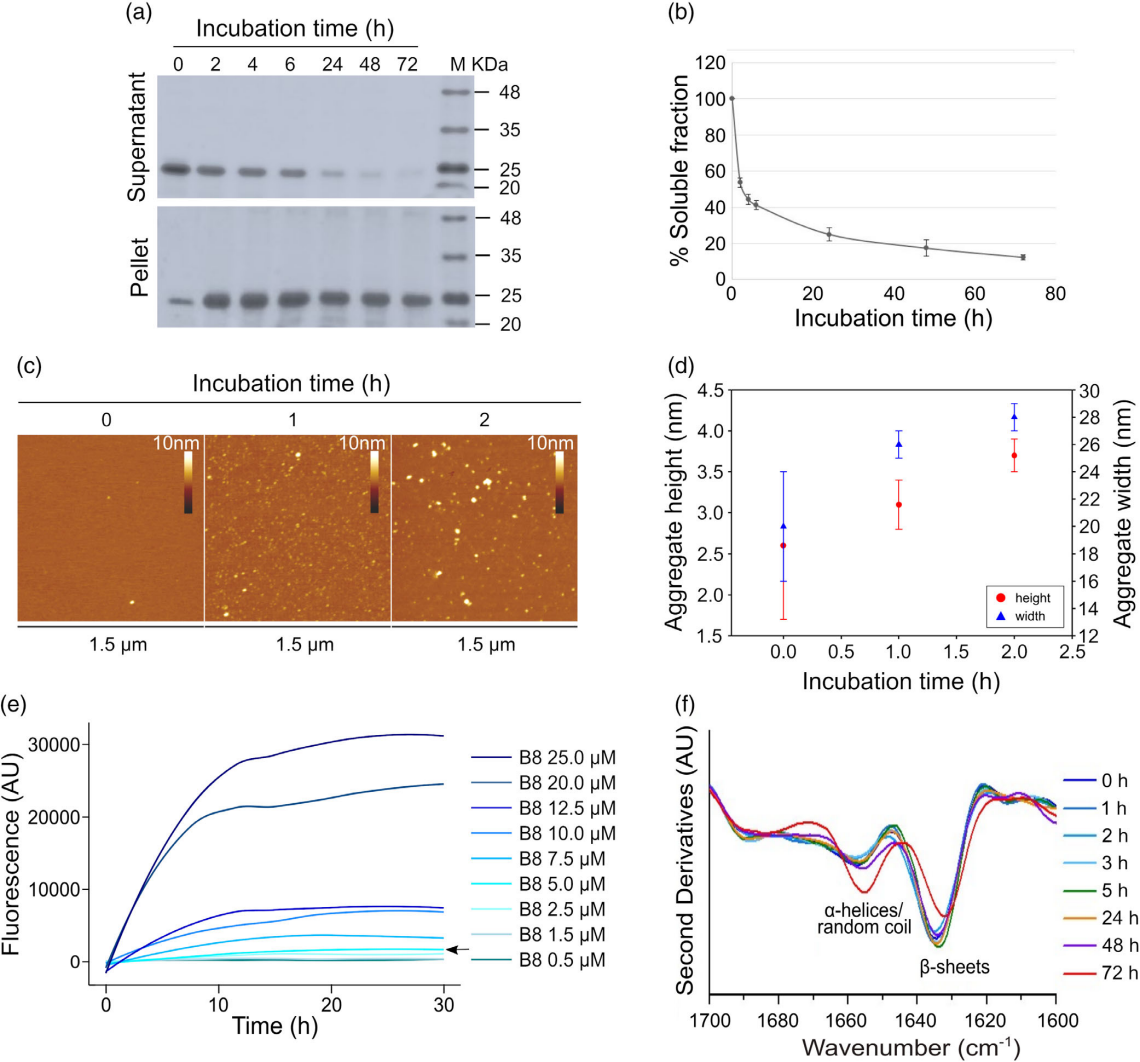
HspB8 self‐assembling. (a) Solubility assay of HspB8: 20 μM of freshly purified protein was incubated in PBS supplied by protease inhibitors at 37°C and aliquots of 20 μL at different times (0, 2, 4, 6, 24, 48, and 72 h) were centrifuged and both supernatants and pellets were subjected to SDS‐PAGE (14%). Gels were stained with EZBlue gel staining solution and scanned at 700 nm with Odyssey Fc System (LICOR). (b) The densitometry analysis was performed with the Image Studio software (LI‐COR). (c) Tapping mode AFM images (height data) of HspB8 incubated in PBS at *t* = 0 and after 1 and 2 h. *Z* range 10 nm. Scan size 1.5 μm. (d) The aggregate mean height (red circles) and width (blue triangles) measured from cross sections of topographic AFM images are reported as a function of the incubation time. The number of analyzed aggregates was (0 h) 13; (1 h) 60; (2 h) 63. The measured widths were corrected for AFM tip size effects. (e) Thioflavin T fluorescence assay: freshly purified HspB8 was incubated at 37°C at different concentrations (μM) in the presence of protease inhibitors. The plot represents ThT fluorescence (in arbitrary units. a. u.) recorded every 30 min with 445/535‐nm excitation/emission filters set. The arrow indicates the 5 μM concentration. Curves are the mean of three independent experiments. with a standard deviation lower than 5%. (f) Second derivatives of the FTIR absorption spectra of HspB8 collected at different incubation times at 37°C. Average spectra from at least three independent experiments are reported in the Amide I band. Time of incubation (in hours) and the assignment of the main components are indicated.

**FIGURE 3:**
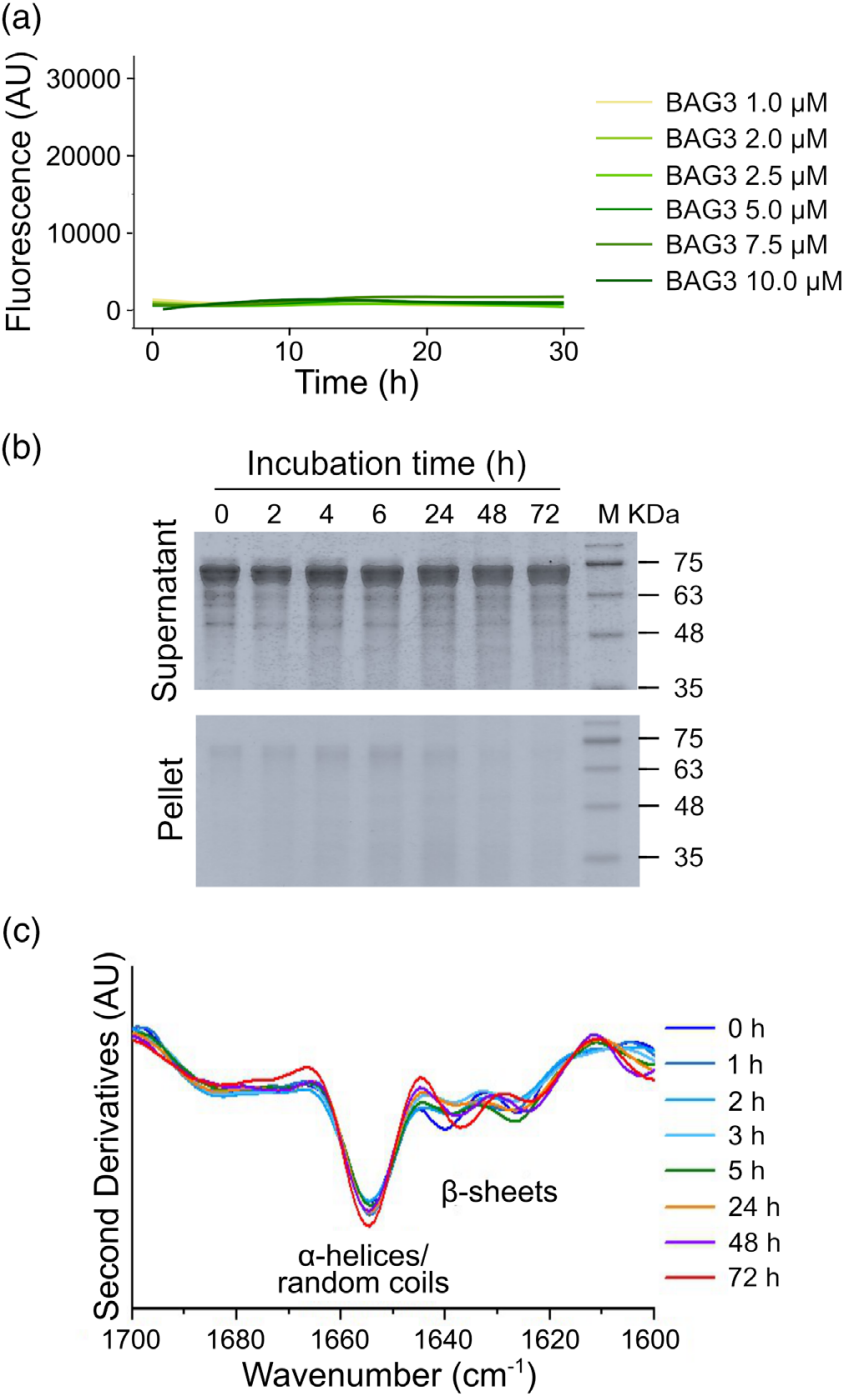
BAG3 self‐assembling. (a) Solubility assay of BAG3: 20 μM of freshly purified protein was incubated in PBS supplied by protease inhibitors at 37°C. Aliquots of 20 μL at different times of incubations (0, 2, 4, 6, 24, 48, and 72 h) were centrifuged and both supernatants and pellets were subjected to SDS‐PAGE (12%). The gels were stained with EZBlue gel staining solution and scanned at 700 nm with Odyssey Fc System (LI‐COR). (b) Thioflavin T fluorescence assay: freshly purified BAG3 was incubated at 37°C at different concentrations (μM) in the presence of protease inhibitors. Plot represents ThT fluorescence (in arbitrary units. a. u.) recorded every 30 min with 445/535‐nm excitation/emission filters set. Curves are the mean of three independent experiments, with a standard deviation lower than 5%. (c) Second derivatives of the FTIR absorption spectra of BAG3 collected at different incubation times at 37°C. Average spectra from at least three independent experiments are reported in the Amide I band. Time of incubation (in hours) and the assignment of the main components are indicated.

Since it is known that some sHsps form oligomeric “reservoirs” (Bova et al., 2000; Bova et al., 2002), solubility assays were carried out to monitor the self‐assembling capability of HspB8. The protein was incubated in PBS at 37°C and aliquots at different incubation times were centrifuged and both supernatants and pellets subjected to SDS‐PAGE (Figure 2a). The densitometry analysis of the supernatants showed a rapid decrease (after 2 h) of the soluble form, while the protein appeared in the pellet, where its amount was kept essentially constant at longer times (Figure 2b). Moreover, the constant presence of protein in the pellet highlights the ability of the aggregated form to resist to the proteolysis differently from the soluble protein that disappears at a longer time. This is reasonably due to the formation of chemical disulfide crosslinking that occurred in our conditions, as demonstrated by the incubation of the HspB8 in the presence of DTT (Figure S1) (Kim et al., 2004; Mymrikov et al., 2012). Noteworthy, the presence of aggregates observed under reducing conditions at the first times of incubation clearly demonstrates that HspB8 oligomerization is independent from chemical disulfide crosslinking (Figure S1). Tapping mode atomic force microscopy (AFM) confirmed the tendency of HspB8 to aggregate. Figure 2c shows representative AFM images of the sample incubated at 37°C at different times. The AFM analysis reveals the presence of globular structures whose size increases with time (Figure 2c,d). Fluorescence analysis with Thioflavin T (ThT) showed that the self‐assembling process is concentration‐dependent, with a lower limit of about 5 μM (Figure 2e). It is important to note that ThT fluorescence is a gold standard for the detection of amyloid aggregates but, nevertheless, ThT also binds non‐amyloid structures, and this binding can be revealed through an increase in its fluorescence (Harel et al., 2008; Rovnyagina et al., 2018). Finally, FTIR spectra recorded at different incubation times at 37°C did not detect important spectral changes until 24–48 h (Figure 2f), although the sample appeared opalescent after 2 h. We should note that FTIR spectroscopy is extensively used for the study of protein misfolding and aggregation since it can be applied to soluble and insoluble materials. Moreover, the Amide I band is particularly sensitive to changes in the protein conformation and, interestingly, to the formation of β‐sheet structures in protein aggregates (Ami & Natalello, 2022; Barth, 2007; Zandomeneghi et al., 2004). According to our data (Figure 2), it can be assumed that HspB8 forms oligomeric “reservoirs” (Figure 2a–e) in “native‐like” conformation (Figure 2f). Otherwise, BAG3 displayed a negligible tendency to aggregate and remained stable until 48 h at 37°C as showed by the solubility and thioflavin T fluorescence assays (Figure 3a,b) and by the FTIR analysis (Figure 3c).

### 2.3 HspB8 and BAG3 interact in “native‐like” conformation

**FIGURE 4:**
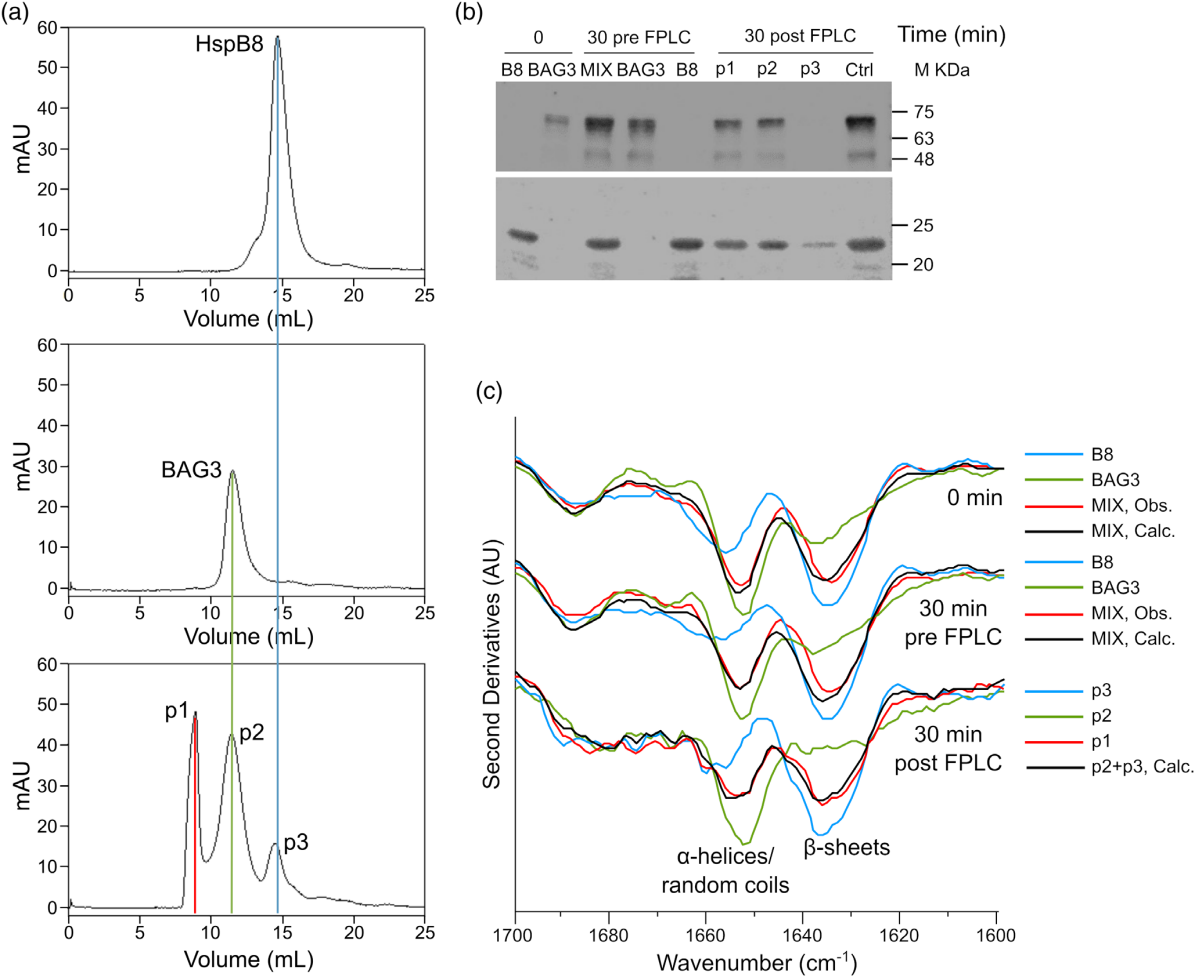
SEC, western blot and FTIR analysis of HspB8 and BAG3 incubated alone and in mixture. (a) SEC analysis: elution profiles of HspB8, BAG3 and the complex obtained by mixing proteins in ratio 1BAG:2HspB8, after incubation of 30 min at 37°C at a concentration of 10 μM each. (b) Western blot analysis: 4 μg of each sample were loaded. In lane 9, 4 μg of BAG3 (top panel) and HspB8 (bottom panel) incubated alone for 30 min (as a control) and after gel filtration was loaded (Ctrl). Immunodetection was performed with anti‐BAG3 monoclonal antibodies (top panel) and with anti‐HspB8 polyclonal antibodies (bottom panel). (c) FTIR analysis: second derivatives spectra of HspB8 and BAG3 incubated alone (blue and green spectra, respectively) and in mixture (red spectra) at time 0 and 30 min before SEC (upper panels) and of peaks 1–3 of SEC (bottom spectra) (obs = observed). In each panel, the black spectrum was obtained by the mathematical sum of the peaks of individual proteins (blue and green spectra) (calc = calculated).

**FIGURE 5:**
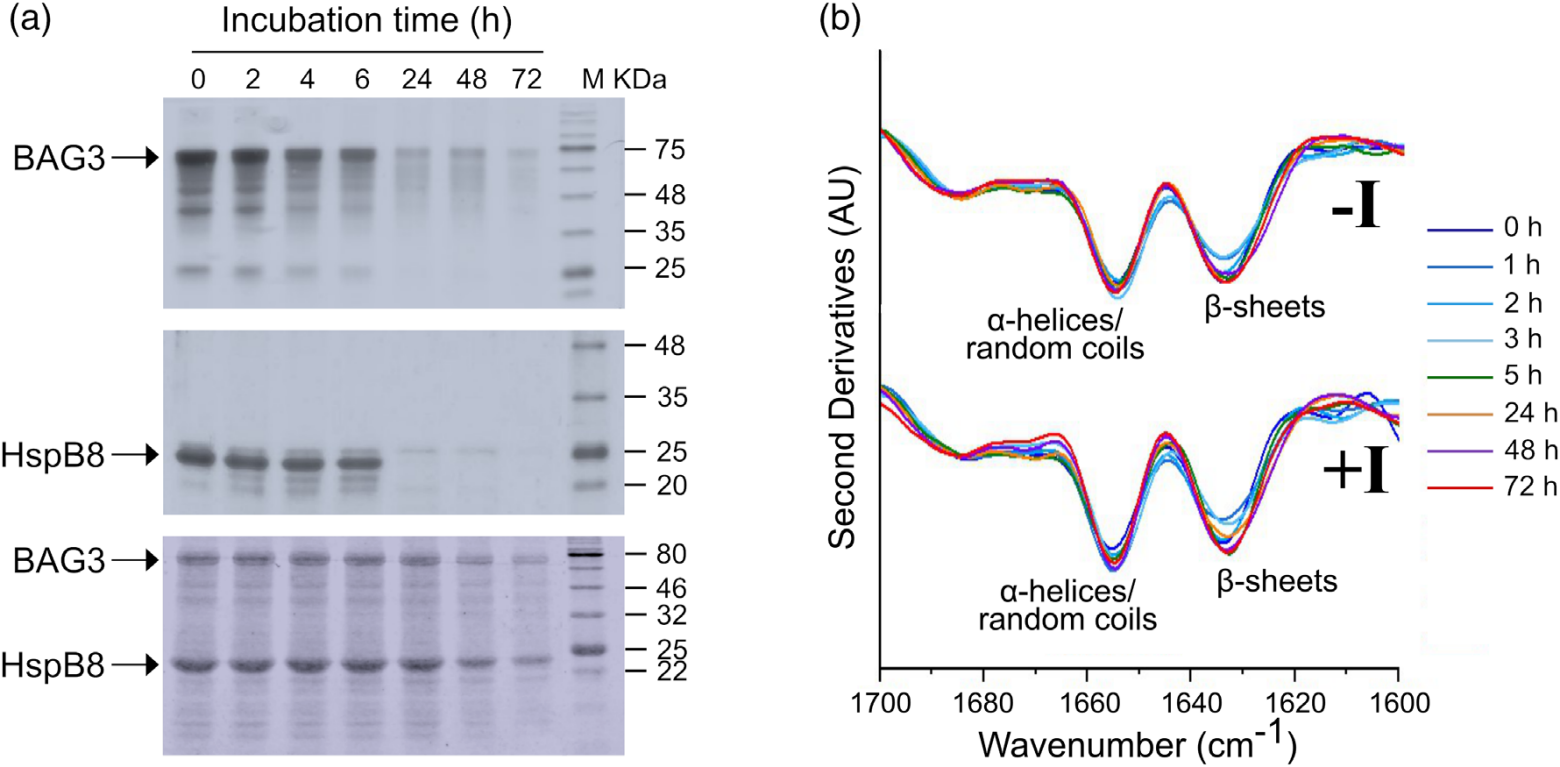
Protein stability of HspB8 and BAG3 incubated alone or in complex and in the absence of protein inhibitors. (a) Solubility assay of HspB8 and BAG3 incubated alone or in association in the absence of protein inhibitors: 20 μM of HspB8, 10 μM of BAG3 and 10 μM of the complex freshly purified were incubated in PBS in absence of protease inhibitors at 37°C and aliquots of 20 μL at different times of incubations (0, 2, 4, 6, 24, 48, and 72 h) were centrifuged and supernatants were subjected to SDS‐PAGE (12% and 14%). The gels were stained with EZBlue gel staining solution and scanned at 700 nm with Odyssey Fc System (LI‐COR). (b) Second derivatives of the FTIR absorption spectra of BAG3:HspB8 mixture collected at different incubation times at 37°C in the absence (−I) and in the presence (+I) of protease inhibitors. Time of incubation (in hours) and the assignment of the main components are indicated.

To further understand the mode of HspB8 and BAG3 interaction, pure proteins were incubated for 30 min at 37°C, both in isolation and as a mixture at a BAG3:HspB8 1:2 ratio (in keeping with the stoichiometry reported in literature (Shemetov & Gusev, 2011)). SEC analysis performed on the samples after incubation highlighted the propensity of HspB8 and BAG3 to form a complex with respect to auto‐assemble (Figure 4a). Western blot analysis performed with specific antibodies anti HspB8 and BAG3 on the different peaks obtained after SEC confirmed the presence of both proteins in the peak corresponding to the complex (p1) (Figure 4b). The IR spectra collected at time 0 and 30 min from the experimental mixture of BAG3 and HspB8 (red spectra in Figure 4c) display two main components respectively assigned to α‐helical structures (related to BAG3) and β‐sheets (related to HspB8). The comparison of these spectra with the computational spectra obtained by the sum of those of the pure proteins (black spectra in Figure 4c) revealed minor spectral changes for the proteins in the mixture. The FTIR analysis, performed for the different peaks obtained after SEC, confirmed the presence of both proteins in the peak corresponding to the complex and indicated that the spectrum of the complex (p1 in Figure 4c) is very similar to that obtained by the mathematical sum of those from the peak of the individual proteins (Figure 4c). These FTIR results (Figure 4c) strongly support the hypothesis of a “native‐like” interaction between the two proteins. Moreover, both proteins were unstable and subject to proteolysis when incubated in isolation at 37°C in the absence of protease inhibitors (Figure 5a) (unlike that shown in Figure 2a,b where proteins were incubated in the presence of inhibitors), while the complex appeared to be stable up to a week under the same conditions (Figure 5a,b). In particular, in Figure 5b the second derivatives of the IR absorption spectra of BAG3:HspB8 mixture collected at different incubation times in the absence and in the presence of protease inhibitors were reported. Considering the relative area of the p1, p2 and p3 peaks (Figure 4a), the observed spectral features of the mixture (Figure 5b) agree with the result reported in Figure 4c (bottom spectra) and confirm that BAG3 and HspB8 interact without major conformational changes. Therefore, this interaction does not significantly change the overall secondary structures but increases the proteolysis resistance by hiding cleavage sites and/or by affecting the protein tertiary structures.

### 2.4 HspB8 tightly binds BAG3 in vitro

**FIGURE 6:**
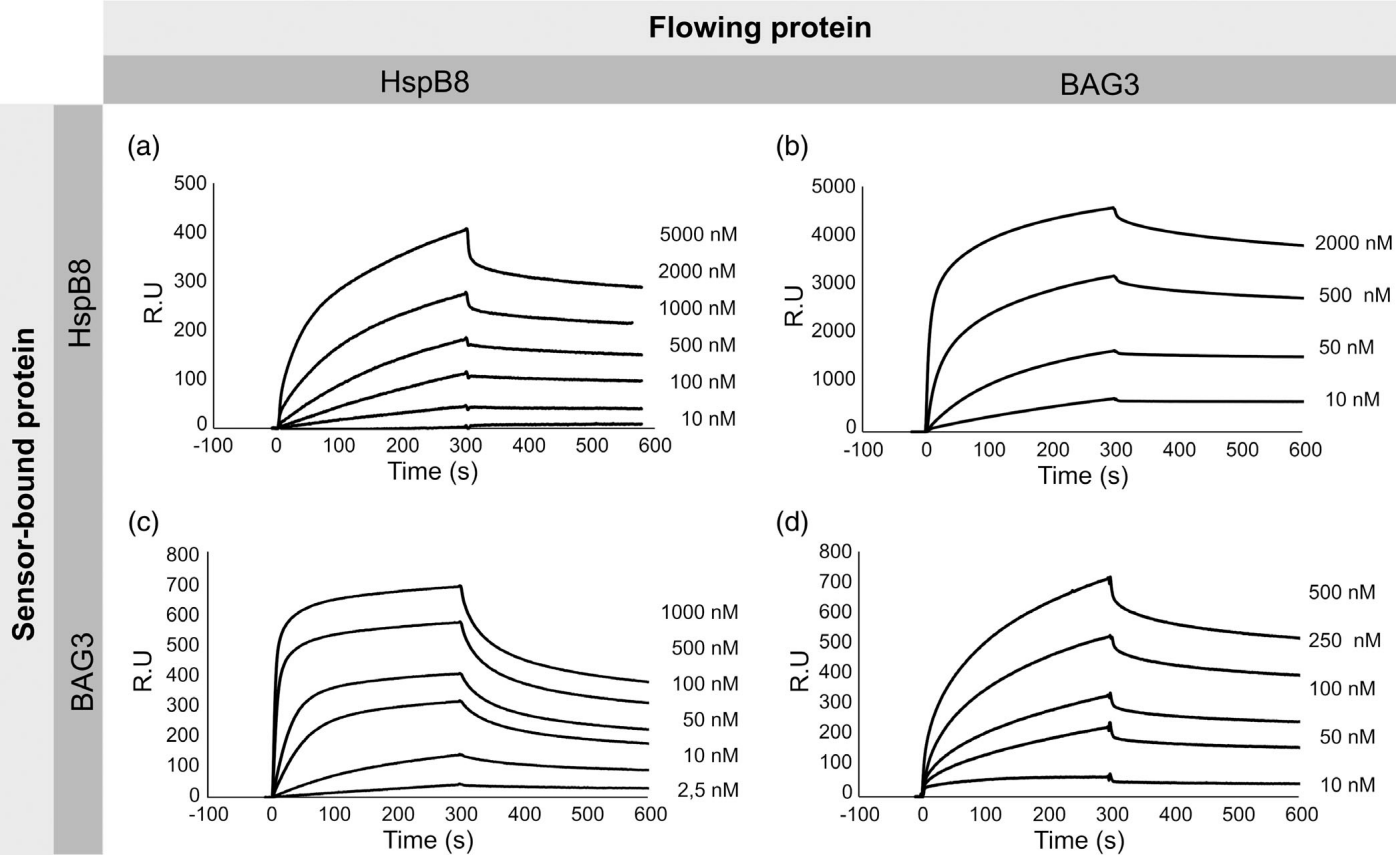
SPR analysis of HspB8 and BAG3 self‐assembly and of the complex. Association/dissociation kinetics for the binding between HspB8 immobilized on the sensor chip and the indicated concentrations of HspB8 (a) and BAG3 (b) flowed onto the sensor chip surface.

Surface plasmon resonance (SPR) analysis was performed to determine the binding affinity of HspB8‐HspB8, BAG3‐BAG3, and HspB8‐BAG3 interactions. In particular, the real time association and dissociation of HspB8 or BAG3 to HspB8 coupled directly to the sensor chip CM5 were determined by monitoring the binding and release of both proteins from the chip (Figure 6a,b). The *K*_D_ obtained displayed a much higher propensity of HspB8 to bind BAG3 (2.4 nM), rather than to auto‐assemble (190 nM) (Table 1). The *K*_D_ binding to BAG3 was also validated using a chip where BAG3 was immobilized and HspB8 was made to flow (4.5 nM) (Figure 6c and Table 1).

**TABLE 1:** Association/dissociation kinetics and affinity values of HspB8‐HspB8, BAG3‐BAG3, and HspB8‐BAG3 interactions.

Sensor‐bound protein	Flowing protein	*k*_on_ (1/nMs)	*k*_off_ (1/s)	*K*_A_ (1/nM)	*K*_D_ (nM)
HspB8	HspB8	(2.50 ± 0.04)*10^−6^	(4.8 ± 1.2)*10^−4^	(5.2 ± 1.3)*10^−3^	(1.9 ± 0.5)*10^2^
	BAG3	(9.3 ± 4.7)*10^−5^	(2.20 ± 0.07)*10^−4^	(4.2 ± 2.3)*10^−1^	2.4 ± 1.4
BAG3	HspB8	(1.3 ± 0.9)*10^−4^	(5.6 ± 3.3)*10^−4^	(2.2 ± 0.3)*10^−1^	4.5 ± 0.7
	BAG3	(1.6 ± 0.8)*10^−5^	(3.0 ± 1.8)*10^−4^	(5.3 ± 0.7)*10^−2^	(1.9 ± 0.2)*10^1^

*Note*: Averages and standard deviations of the association/dissociation kinetics and affinity values are indicated (*n* = 2).

### 2.5 HspB8 alone and in the complex affects Josephin domain aggregation

**FIGURE 7:**
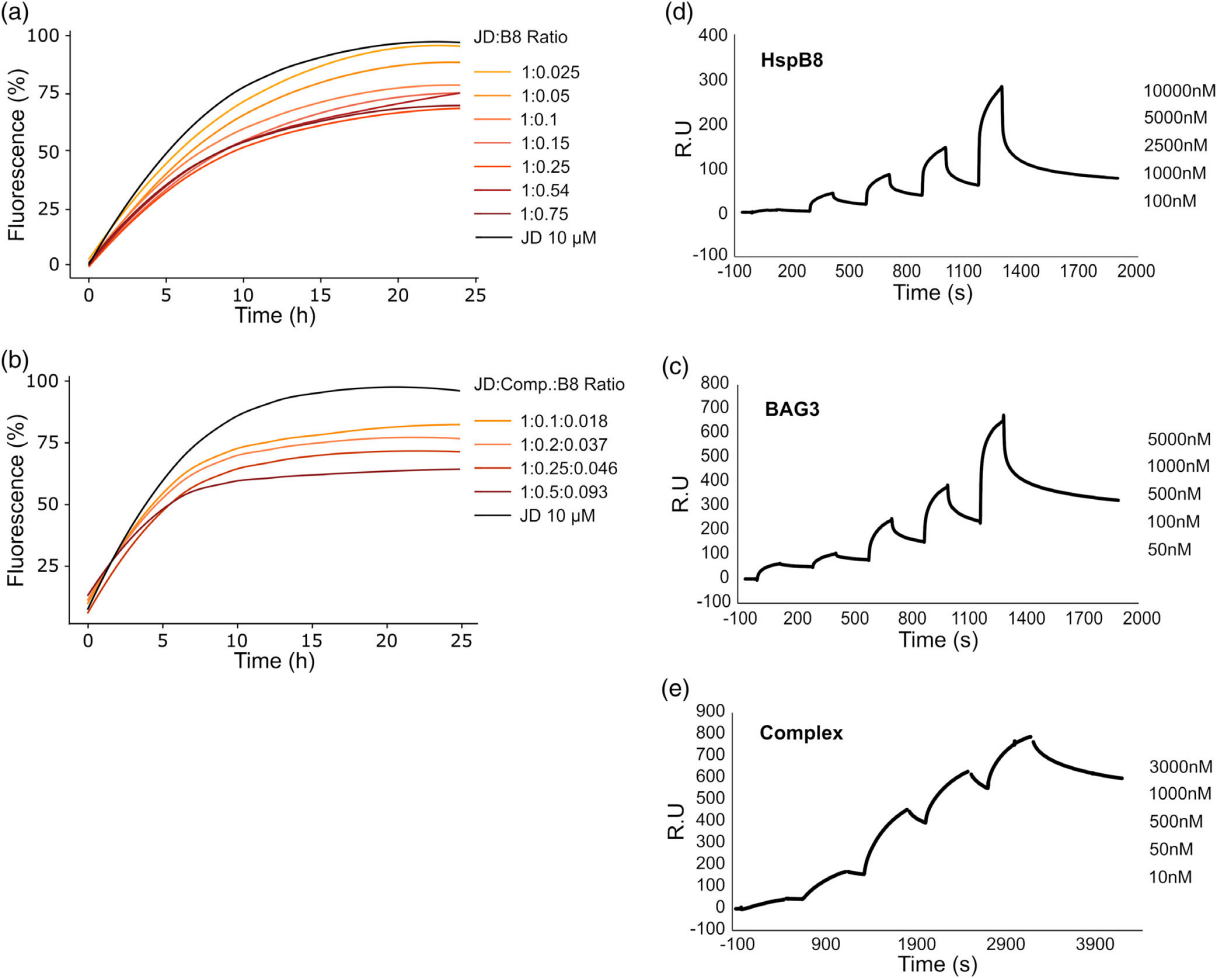
Analysis of HspB8 chaperone‐like activity and binding activity versus JD alone and in the association with BAG3. (a) ThT fluorescence assay: freshly purified JD (10 μM) was incubated at 37°C alone and in the presence of HspB8 or HspB8‐BAG3 complex at different ratio. Plots represent the percentage of the highest fluorescence value reached at each concentration normalized respect to JD. Curves are the mean of three independent experiments. with a standard deviation lower than 5%. (c–e) SPR of the Single Cycle Kinetics (SCK) sensograms showing the binding between JD immobilized on the sensor chip and the indicated concentrations of BAG3 (c). HspB8 (d). HspB8‐BAG3 complex (e) flowed onto the sensor chip surface.

Since it is well known that HspB8 is able to affect protein aggregation in neurodegenerative diseases (Carra, Seguin, Lambert, & Landry, 2008a; Chowdary et al., 2004; Kim et al., 2004; Sanbe et al., 2007), chaperone‐like activity of HspB8 was assessed on the Josephin domain (JD), structured region that triggers the aggregation process of ataxin‐3 (ATX3) and displayed amyloidogenic properties when incubated alone (Masino et al., 2011; Robertson et al., 2010). The assay was performed by incubating freshly purified JD with HspB8 at different molar ratios and monitoring the fluorescence signal of ThT. The resulting profiles showed that HspB8 is able to inhibit JD aggregation, with a maximal reduction of 35% at a molar ratio protein JD:HspB8 of 4:1 (Figure 7a). At higher ratios, a decrease in HspB8 inhibitory ability was observed, likely due to its own aggregation, in according with the results reported in Figure 2e. In particular, the complex displayed higher activity with respect to HspB8 alone. In fact, the maximum percentage of inhibition (40%) of the complex was obtained at the JD:complex ratio of 1:0.5, which under our experimental conditions is equivalent to a JD:HspB8 ratio of 1:0.093 (Figure 7b). Noteworthy, HspB8 alone has only 20% of activity at the same JD:HspB8 ratio (Figure 7a). This result is in agreement with the lower *K*_D_ binding value obtained immobilizing JD on chip and flowing different concentrations of the HspB8‐BAG3 complex (*K*_D_ = 73 nM) with respect to HspB8 and BAG3 alone (*K*_D_ = 8.1 μM and 5.4 μM, respectively) (Figure 7c–e and Table 2).

**TABLE 2:** Association/dissociation kinetics and affinity values of HspB8, BAG3, and HspB8‐BAG3 complex versus the Josephin domain.

Sensor‐bound protein	Flowing protein	*k*_on_ (1/nMs)	*k*_off_ (1/s)	*K*_A_ (1/nM)	*K*_D_ (nM)
JD	BAG3	(2.60 ± 0.07)*10^−7^	(1.40 ± 0.07)*10^−3^	(1.9 ± 0.6)*10^−4^	(5.4 ± 1.8)*10^3^
	HspB8	(7.4 ± 3.8)*10^−8^	(6.0 ± 1.9)*10^−4^	(1.2 ± 0.3)*10^−4^	(8.1 ± 1.8)*10^3^
	Complex	(3.3 ± 0.3)*10^−6^	(2.4 ± 0.5)*10^−4^	(1.4 ± 0.2)*10^−2^	(7.3 ± 0.9)*10^1^

*Note*: Averages and standard deviations of the association/dissociation kinetics and affinity values are indicated (*n* = 2).

## 3 DISCUSSION

An efficient protein quality control concerning the cooperative action of chaperones and co‐chaperones is fundamental to counteract the aggregation of misfolded proteins that are responsible of several age‐related neurodegenerative disorders (Balchin et al., 2016). One major gap in the chaperone network knowledge is how sHsps link to the other major components. In this work, we provide a deeper understanding of the tendency of HspB8 to auto‐assemble or interact with BAG3 forming a complex that is involved in protein homeostasis (Carra et al., 2009; Carra, Seguin, & Landry, 2008b). We initially characterized the two proteins from a structural point of view, confirming the presence of both disordered and folded domains (Figure 1). Then, we investigated the propensity of both proteins to self‐assemble, confirming the HspB8 tendency to form oligomeric ensembles above a certain concentration (higher than 5 μM) within a relatively short time (Figure 2a–d). Noteworthy, our analyses revealed that HspB8 assembles in oligomers (by AFM) with “native‐like” conformation (by FTIR) (Figure 2e). The results are in accordance with the idea that sHsps form dynamic oligomeric complexes that act as reservoirs of the active dimeric form, which is released in the presence of stress conditions (Santhanagopalan et al., 2018). Otherwise, BAG3 displayed a negligible tendency to aggregate (Figure 3). The apparent discrepancy with the *K*_D_ values of auto‐assembling observed by SPR analysis (lower in the case of BAG3 with respect to HspB8) could be ascribed to the technical design (in which one partner is immobilized) and to the short time of SPR analysis that permits to obtain *K*_D_ values which refer to the formation only of small homo‐ and hetero‐oligomers. The tendency of HspB8 to auto‐assemble or to bind BAG3 might be related to its instability, observed in vivo (Carra, Seguin, Lambert, & Landry, 2008a), and confirmed by our results showing that both HspB8 and BAG3 displayed a strong sensitivity to proteolytic degradation when incubated alone (Figure 4). This is also reasonably due to their partially disordered nature. Differently, FTIR analysis clearly showed the conformational stability of the purified complex incubated in the absence of protease inhibitors for several days (Figure 4). These results supplement the early characterizations of Shemetov on HspB8‐BAG3 complex in which the increase in thermal stability and resistance to proteolysis of the complex were highlighted (Shemetov & Gusev, 2011). The authors also suggested that the interaction of the two proteins leads to partial ordering of their structures (Shemetov & Gusev, 2011). Our FTIR analysis performed on HspB8‐BAG3 complex purified by SEC clearly demonstrated the “native‐like” conformation of both proteins when associated (Figure 3), without detectable induction of new α‐helical and β‐sheet structures. One point to clarify is the tendency of HspB8 to exist in complex with BAG3 or in the reservoir in vivo. There is evidence that HspB8 formed tight complexes with BAG3 in mammalian cell (Carra, Seguin, Lambert, & Landry, 2008a). In addition, the lack of the conserved I/V‐X‐I/V motif, responsible for oligomeric assembly in the C‐terminal of HspB8, as well for HspB6, leads to the formation of small oligomers unlike the other sHsps (Kim et al., 2004). The significant difference in *K*_D_ values obtained by SPR analysis clearly shows the higher propensity of HspB8 to bind BAG3 than to homo‐oligomerize (2.4 nM vs. 190 nM respectively, Figure 6 and Table 1). This was already shown in SEC experiments where, after 30 min of incubation, the appearance of the p1 peak (due to the complex formation, Figure 4a) occurred only in the presence of BAG3. These results, in addition to the above literature data, strongly support the model that HspB8 could be an obligate partner of BAG3 in vivo (Carra, Seguin, Lambert, & Landry, 2008a). If so, it is even more important to understand its contribution to the complex activity. As already mentioned, HspB8 seems to be involved in several physiological mechanisms. HspB8 displays chaperone‐like activity in preventing DTT‐induced insulin and thermal‐induced citrate synthase aggregation in vitro (Chowdary et al., 2004) and prevents aggregation of amyloid proteins, such as CryAB‐amyloid (Sanbe et al., 2007) and tau (Webster et al., 2020). Its chaperone activity was also observed in cells expressing polyglutamine protein Htt43Q (Carra et al., 2005) and human brain pericytes where HspB8 overexpression reduced amyloid β 1–40 peptide aggregation and cytotoxicity (Wilhelmus et al., 2006). Moreover, HspB8 ensures the proteolytic degradation of misfolded proteins such as in the case of the HspB8 mediated‐cardiac hypertrophy (Hedhli et al., 2008). Finally, in the CASA complex HspB8 contributes to the recognition and elimination of misfolded proteins through the autophagosome–lysosome pathway (Arndt et al., 2010). In this complex scenario, at first we demonstrated the capability of HspB8 to inhibit the aggregation of the Josephin domain, the structured part of the ataxin‐3 protein (ATX3) that triggers the earliest steps of the expanded ATX3 fibrillation and the onset of the Spinocerebellar Ataxia type 3 (Masino et al., 2011; Robertson et al., 2010). Noteworthy, we observed that the inhibitory activity decreases when HspB8 concentration reaches and exceeds the limit beyond which the protein begins to self‐assemble (Figure 7a and Figure 2e). It is possible that a competition for the binding between HspB8‐JD and HspB8 monomers/dimers occurs, and this could be justified by the difference between the two *K*_D_ (8100 nM and 190 nM, respectively) (Tables 1 and 2). At this point, we investigated if this activity could be maintained in association with BAG3, taking into account our results that their interaction is in native‐like conformation (Figure 4). Remarkably, we demonstrated that the HspB8‐BAG3 complex displays more chaperone‐like activity, in term of capability to prevent the JD aggregation, at the same JD:HspB8 ratio (Figure 7a,b). In keeping with this, the complex displays a lower *K*_D_ value for JD binding with respect to the single proteins and this may be due to the binding contribution of BAG3. These results further support that BAG3 is not a “passive” scaffolding protein, but a component that can influence the stability and the activity of its chaperone partners (Rauch et al., 2017). Several works reported an activity of the HspB8‐BAG3 complex uncoupled from the Hsp70/Hsc70 chaperone system, suggesting a different mode of action with respect to the CASA process (Carra et al., 2009; Carra et al., 2010; Carra, Seguin, Lambert, & Landry, 2008a; Carra, Seguin, & Landry, 2008b; Guilbert et al., 2018; Seidel et al., 2012). In cells, HspB8 cooperates with BAG3 to stimulate autophagy in an eIF2α‐dependent manner and facilitates the clearance of aggregate‐prone proteins (Carra et al., 2009; Carra et al., 2010; Carra, Seguin, Lambert, & Landry, 2008a; Carra, Seguin, & Landry, 2008b). The complex is also involved in the remodeling of actin‐based mitotic structures that guide spindle orientation and proper chromosome segregation during mitotic cell division (Guilbert et al., 2018). Although the specific mechanism has not yet been established, it has been proposed that HspB8 provides the substrate recognition capacity whereas BAG3 facilitates the interaction of the substrate with the p62 ubiquitin adaptor. Therefore, we can assume that the chaperone‐like activity shown by the complex could have a role in its physiological functions. It would be interesting to assess whether the chaperone‐like activity of HspB8 is retained even in presence of Hsp70/Hsc70. In this case, we could speculate that HspB8 could not only bind the misfolded proteins and prevent the aggregation but could keep them in a state more accessible to the Hsp70 activity, without energy consumption by the cell.

In conclusion, this current study clearly demonstrates that HspB8 self‐assembles and interacts with BAG3 in native‐like conformation and displays a chaperone‐like activity versus JD both alone and in association with BAG3. Carra and coauthors have shown that overexpression of human HspB8 protects against mutated ataxin‐3‐induced eye degeneration in an in vivo Drosophila model of SCA3 (Carra et al., 2010). Our in vitro results strongly suggest that chaperone‐like activity of HspB8 alone or in the complex could affect ataxin‐3 aggregation acting on JD oligomerization. All this considered, we can argue that HspB8 may be a good target for therapeutic intervention in SCA3.

## 4 MATERIALS AND METHODS

### 4.1 HspB8, BAG3, Josephin domain cloning and expression

The HspB8 gene cloned in pGEX‐6P‐1 vector (GE Healthcare LifeSciences, Little Chalfont, England) was kindly provided by Serena Carra (University of Modena‐Reggio Emilia, Italy) and the protein was expressed in *Escherichia coli* BL21‐CodonPlus(DE3)‐RIL strain (argU (AGA, AGG), ileY (AUA), leuW (CUA)) as N‐terminal glutathione S‐transferase (GST) fusion protein. BAG3 gene sequence (NCBI code number NM_004281.3) was synthetized and cloned in pQE‐30 plasmid (Qiagen, Hilden, Germany) between BamHI and HindIII sites. His‐tagged protein was expressed in *E. coli* M15 (pREP4) strain (F^−^, Φ80ΔlacM15, thi, lac^−^, mtl^−^, recA^+^, KmR). The Josephin domain (JD, 182 aa) is the structured part of the ataxin‐3 proteins that does not contain the poly Q stretch. The JD‐encoding gene was cloned in pET21‐a vector and the protein was expressed in *E. coli* BL21 Tuner (DE) pLacI (*E. coli* B F^−^ ompT hsdSB (rB^−^ mB^−^) gal dcm lacY1 (DE3) pLacI (CamR); Novagen, Germany) and purified as previously described (Bonanomi et al., 2015).

### 4.2 HspB8, BAG3 and JD purification

Cells were grown at 37°C in LB low salt‐ampicillin medium for the strains expressing HspB8 and JD while in 2TY medium supplemented with ampicillin and kanamycin for BAG3 strain. HspB8 and JD protein expression was induced with addition of 0.5 mM IPTG at OD_600_ 0.8 for 1 h and 3 h, respectively. BAG3 protein expression was induced with 1 mM IPTG at OD_600_ 0.8 at 30°C for 3 h and 45 min respectively. JD purification was performed as previously reported (Bonanomi et al., 2015). For HspB8 purification, about 2.5 g of cells was pelleted from 1 L of culture. To obtain crude extracts, cells were resuspended in 5 mL/g_cell_ of lysis buffer (25 mM potassium phosphate, pH 7.2, 0.15 M NaCl, 0.5 mM PMSF, 5 mM DTT, 100 mM MgCl_2_, 1 mg/mL lysozyme, SigmaFAST™ Protease Inhibitor Cocktail EDTA Free containing AEBSF 2 mM, Phosphoramidon 1 μM, Bestatin 130 μM, E‐64 14 μM, Leupeptin 1 μM, Aprotinin 0.2 μM, Pepstatin 10 μM) (Sigma–Aldrich, St. Louis, MO, USA) and sonicated three times for 30 s, amplitude 20%. 1% Triton‐X‐100 and DNase (0.2 mg/g_cell_) were then added, and the samples were further incubated for 30 min at room temperature. Finally, they were centrifuged for 30 min at 47,900 g. The supernatants were loaded onto a Glutathione Sepharose™ 4 Fast Flow affinity column (1 mL bed volume), previously equilibrated with 10 volumes of PBS buffer (25 mM potassium phosphate, pH 7.2, 0.15 M NaCl) with 2 mM PMSF and incubated for 40 min at 4°C on wheel. After washing with 10 bed volume of cleavage buffer (50 mM Tris/HCl pH 7.2, 150 mM NaCl, 1 mM EDTA, 1 mM DTT), purified HspB8 was obtained by incubation overnight with 100 U/mL_resin_ of PreScission Protease (PreScission™ Protease, GE Healthcare LifeSciences, Little Chalfont, England) at 4°C on wheel. The protein was then eluted with PBS buffer, while GST‐PreScission Protease, remained bound to the resin. Subsequently, to remove the GST contamination, the eluted protein was further load onto 0.5 mL of resin, incubated for 40 min at 4°C on rotating wheel, and collected by gravity. For BAG3 purification, about 20 g of cells from 5 L were resuspended in 5 mL/g_cell_ of lysis buffer (25 mM potassium phosphate, pH 7.2, 0.15 M NaCl, 2 mM PMSF, 10 mM imidazole, 10% glycerol, 1 mM 2‐mercaptoethanol, 1 mg/mL lysozyme, SigmaFAST™ Protease Inhibitor Cocktail EDTA Free) (Sigma–Aldrich, St. Louis, MO, USA) and sonicated three times for 1 min, amplitude 20%. DNase (0.2 mg/g_cell_) is added, and the samples were further incubated for 30 min at room temperature. Finally, they were centrifuged for 30 min at 47,900 g. The supernatant was loaded onto HisPur™ Cobalt Resin (Thermo Fisher Scientific, Rockford, IL, USA) and washed with 10 bed volumes of wash buffer (25 mM potassium phosphate, pH 7.2, 150 mM NaCl, 2 mM PMSF, 10 mM imidazole, 10% glycerol, 1 mM 2‐mercaptoethanol). The bound protein was then eluted with elution buffer (25 mM potassium phosphate, pH 7.2, 150 mM NaCl, 150 mM imidazole, 10% glycerol, 1 mM 2‐mercaptoethanol).

Before each experiment, proteins were loaded onto a Superose 12 10/300 GL gel filtration column (GE Healthcare, Life Sciences, Little Chalfont, England), pre‐equilibrated with PBS buffer. Elution was performed at a flow rate of 0.5 mL/min in the same buffer. To avoid the BAG3 degradation, the PBS buffer was supplied with SigmaFAST™ Protease Inhibitor Cocktail EDTA Free (Sigma–Aldrich, St. Louis, MO, USA). A calibration curve was prepared by plotting elution volume parameters of a set of standard proteins against the logarithm of their molecular weights. Standards employed at 1 mg/mL: Apoferritin (PM: 450 KDa), Alcohol Dehydrogenase (PM: 150 kDa), bovine serum albumine (67 kDa), bovine β‐lactoglobulin (35 kDa), bovine cytochrome C (12.7 kDa) (Sigma–Aldrich, St. Louis, MO).

Fractions were collected and protein content determined using Coomassie brilliant blue G‐250 (Thermo Scientific Rockford, IL, USA) and bovine serum albumin as a standard protein.

### 4.3 HspB8‐BAG3 complex purification

The purified proteins were incubated alone or in a mixture with the ratio BAG3:HspB8 1:2 ratio for 30 min at 37°C in agitation. SEC chromatography was performed to isolate the proteins and the complex. The presence of the different proteins in the peaks was revealed by western blot analysis using specific antibodies.

### 4.4 SDS‐PAGE and densitometry analysis of soluble protein fraction

Freshly purified HspB8 and BAG3 (20 μM) were incubated at 37 °C in PBS buffer with and without 15 mM DTT; aliquots at different incubation times (0 h, 2 h, 4 h, 6 h, 24 h, 48 h, 72 h) were centrifuged at 12,000 rpm for 15 min, both supernatants and pellets were subjected to SDS‐PAGE (12%). Pellets were resuspended in 10 μL of Sample Buffer (50 mM Tris–HCl pH 6.8, 0.4% SDS, 4% Glycerol, 0.141 M 2‐mercaptoethanol) and loaded on the gels. 5 and 10 μL of supernatant were loaded for BAG3 and HspB8 respectively. The gels were stained with EZBlue™ Gel Staining Reagent (Sigma–Aldrich, St. Louis, MO, USA), scanned at 700 nm with Odyssey Fc System (LI‐COR Biosciences, Lincoln, NE, USA) and the densitometry analysis were performed with the Image Studio software (LI‐COR Biosciences, Lincoln, NE, USA).

### 4.5 Western blot analysis

Western blot was performed according to standard procedures using Odyssey Nitrocellulose Membrane (LI‐COR Biosciences, Lincoln, NE, USA). Rabbit monoclonal anti‐BAG3 antibody [EPR3515] ab92309 (Abcam, Cambridge, UK) at a 1:5000 dilution, rabbit polyclonal anti‐HspB8, kindly provided by Angelo Poletti (University of Milan, Italy) at a 1:1000 dilution and the secondary antibody Donkey anti‐Rabbit IRDye (LI‐COR Biosciences, Lincoln, NE, USA) at a 1:15,000 dilution were employed. Fluorescence was scanned at 800 nm with Odyssey Fc System (LI‐COR Biosciences, Lincoln, NE, USA).

### 4.6 Thioflavin T assay

Thioflavin T (ThT) assay was performed to monitor the aggregation process of HspB8 and BAG3 (Harel et al., 2008; Rovnyagina et al., 2018). This assay measures changes of fluorescence intensity of ThT upon binding to protein aggregates. Freshly purified HspB8, BAG3 and the complex at different concentrations were incubated in PBS buffer at 37°C in the presence of 20 μM ThT (Sigma–Aldrich, St. Louis, MO, USA). The complex used in ThT assay was obtained by SEC (p1 peak of Figure 4a) and its concentration was calculated considering a molecular weight of 118 KDa (2HspB8:1BAG3). The fluorescence was measured in clear‐bottomed black ViewPlate®‐96 F TC (Perkin Elmer, MA, USA), using a VICTOR TM X3 Multilabel Plate Reader (Perkin Elmer, MA, USA). Excitation and emission wavelengths were 445 nm and 535 nm, respectively. Readings were carried out from the bottom of the plates with no shaking and recorded every 30 min for 72 h. 100 μL of mineral oil and a lid were used to prevent evaporation. The ThT data were normalized by plotting the change in ThT fluorescence (in arbitrary units, a. u.), determined as (*F*–*F*0), where *F* is the mean ThT fluorescence reading from technical triplicate sample and *F*0 is the mean ThT fluorescence reading from technical triplicate of the reference sample (ThT alone). The curves in the pictures are the mean of three independent experiments. Standard deviations never exceeded 5%.

ThT assay was also used to follow the JD aggregation. In this case, ThT fluorescence was monitored incubating 10 μM JD alone or in the presence of different ratio of HspB8, BAG3 and the complex. The two proteins and the complex were purified in SEC in the absence of protein inhibitors in order to not alter the JD kinetics of aggregation. We have assayed HspB8 activity alone and in the complex.

In our experimental conditions, we are not able to test BAG3 activity on JD, since the addition of protease inhibitors, necessary for its stability, affects JD aggregation.

### 4.7 Circular dichroism analysis

CD spectra in the far‐UV region (Kelly et al., 2005; Natalello et al., 2012) of HspB8 (at 5 μM) and BAG3 (at 2.5 μM) were collected by the Jasco J‐815 (Jasco Corp., Tokyo, Japan) spectropolarimeter in a 0.1‐cm path‐length quartz cell and averaged over three scans. CD spectra are presented after buffer subtraction and smoothing. The prediction of the protein secondary structure from the circular dichroism spectra was obtained by using the BeStSel web server (Micsonai et al., 2015).

### 4.8 FTIR spectroscopy analysis

For FTIR measurements in attenuated total reflection (ATR), 2 μL of the protein preparations were deposited on the diamond plate of the single reflection ATR device (Quest, Specac, USA) and the spectra were recorded after solvent evaporation as previously described (Ami & Natalello, 2022). The Varian 670‐IR spectrometer (Varian Australia Pty Ltd., Mulgrave VIC, AU) was employed under the following conditions: 1000 scan coadditions, 2 cm^−1^ spectral resolution, 25 kHz scan speed, triangular apodization, and nitrogen‐cooled Mercury Cadmium Telluride (MCT) detector. After subtraction of the buffer absorption, collected under the same conditions used for the protein samples, and after the subtraction of residual vapor peaks, when necessary, absorption spectra were smoothed using the Savitsky–Golay method (25 points) before the second‐derivative calculation by the Resolutions‐Pro software (Varian Australia Pty Ltd.). Second derivatives can be used to monitor spectral changes if the very same analyses are performed on the original data, providing that the original absorption spectra are of high quality (Dong et al., 1990; Yang et al., 2022; Zhang & Yan, 2005).

### 4.9 Atomic force microscopy analysis

HspB8 was incubated in PBS buffer at 37°C at a concentration of 20 μM. At fixed aggregation times, a 10 μL aliquot was withdrawn and diluted 100 times, and 10 μL of the diluted sample were deposited on a freshly cleaved mica substrate and dried under mild vacuum. Sample dilution in water avoided the formation of salt crystals. AFM images were acquired in tapping mode in air using a Dimension 3100 Scanning Probe Microscope (Bruker, Karlsruhe, Germany) equipped with “G” scanning head (maximum scan size 100 μm) and driven by a Nanoscope IIIa controller (Bruker). Single beam uncoated silicon cantilevers (type OMCL‐AC160TS, Olympus and TESPA_V2, Bruker) were used. The drive frequency was between 290 and 310 kHz, the scan rate between 0.40 and 0.75 Hz. Aggregate height and widths were measured manually from the cross‐sections of topographic AFM images. Widths values were corrected for AFM tip broadening effects as reported previously (D'Andrea et al., 2018). Errors were calculated according to Student's statistics assuming a confidence level of 95%.

### 4.10 Surface plasmon resonance (SPR)

A BIACORE X system (GE Healthcare Life Sciences, Little Chalfont, England) was used to analyze molecular interactions by means of SPR. HspB8 and BAG3 proteins were coupled to a carboxymethylated dextran surface of two different CM5 sensor chips by using amine‐coupling chemistry at surface densities of 7400 and 3400 resonance units, respectively. Appropriate multiple concentrations of the interacting proteins were diluted from the stock protein solution containing PBS in running buffer (10 mM HEPES, pH 7.4, 150 mM NaCl, 3 mM EDTA containing 0.005% [v/v] Surfactant P20) for 5 min. After injection, analyte solutions were replaced by running buffer at a continuous flow rate of 10 μL/min. Surface regeneration was accomplished by injecting 250 mM NaOH for a contact time 1 min. Each sensorgram was subtracted for the response observed in the control flow cell (no immobilized protein) and normalized to a baseline of 0 RU. The interaction rate constants were calculated fitting the sensograms to the model Langmuir Binding 1:1 excluding a short period after injection start and stop and using the BIA evaluation 4.1 SPR kinetic software (GE Healthcare Life Sciences, Little Chalfont, England).

A BIACORE X100 system (Cytiva, Marlborough [HQ], MA, USA) was also used to analyze molecular interactions by means of SPR. JD protein was coupled to a carboxymethylated dextran surface of CM5 sensor chip by using amine‐coupling chemistry at surface density of 3000 resonance units.

Appropriate, multiple concentrations of B8, BAG3 and complex BAG3 + B8 were injected for 120 s (HspB8, BAG3) and for 498 s (BAG3 + HspB8) at 25°C by single cycle kinetics at flow rate of 10 μL/min in running buffer (10 mM HEPES, pH 7.4, 150 mM NaCl, 3 mM EDTA containing 0.005% [v/v] Surfactant P20). After injection, analyte solutions were replaced by running buffer at a continuous flow rate of 10 μL/min. Surface regeneration was accomplished by injecting 250 mM NaOH for a contact time 1 min. Each sensorgram was subtracted for the response observed in the control flow cell (no immobilized protein) and normalized to a baseline of 0 RU. The complex was assumed in the stoichiometric relationship with JD as 1:1 and the interaction rate constants were calculated by fitting the sensograms to the model Langmuir Binding 1:1 and using the BiacoreX100 Evaluation software 2.0.2 (Cytiva, Marlborough [HQ], MA, USA).

## AUTHOR CONTRIBUTIONS

**Barbara Sciandrone**: Investigation, data curation, formal analysis, visualization, writing—original draft, writing—review & editing; **Diletta Ami**: Investigation, data curation, formal analysis, visualization, writing—original draft, writing—review & editing; **Annalisa D'Urzo**: Investigation, data curation, formal analysis, visualization, writing—original draft, writing—review & editing; **Elena Angeli**: Investigation, data curation, formal analysis, visualization, writing—review & editing; **Annalisa Relini**: formal analysis, visualization, resources, writing—review & editing; **Marco Vanoni**: Formal analysis, visualization, resources, writing—review & editing; **Antonino Natalello**: Conceptualization, formal analysis, visualization, resources, supervision, writing—original draft, writing—review & editing; **Maria Elena Regonesi**: Conceptualization, formal analysis, visualization, resources, supervision, writing—original draft, writing—review & editing.

## CONFLICT OF INTEREST STATEMENT

The authors declare no conflicts of interest.

## Supporting information


**Data S1.** Supporting Information
