# Supplementary material for: HspB8 interacts with BAG3 in a “native‐like” conformation forming a complex that displays chaperone‐like activity
Source: Protein Sci. 2023 Jul 1;32(7):e4687. doi: 10.1002/pro.4687 (PMC10273338; doi:10.1002/pro.4687)
Supplement: Supplementary file 1 — Data S1. Supporting Information [file PRO-32-e4687-s001.docx]

**HspB8 interacts with BAG3 in a “native-like” conformation forming a complex that displays chaperone-like activity**

Barbara Sciandrone, Diletta Ami, Annalisa D’Urzo, Elena Angeli, Annalisa Relini, Marco Vanoni, Antonino Natalello, Maria Elena Regonesi

**Supplementary Figure**

**
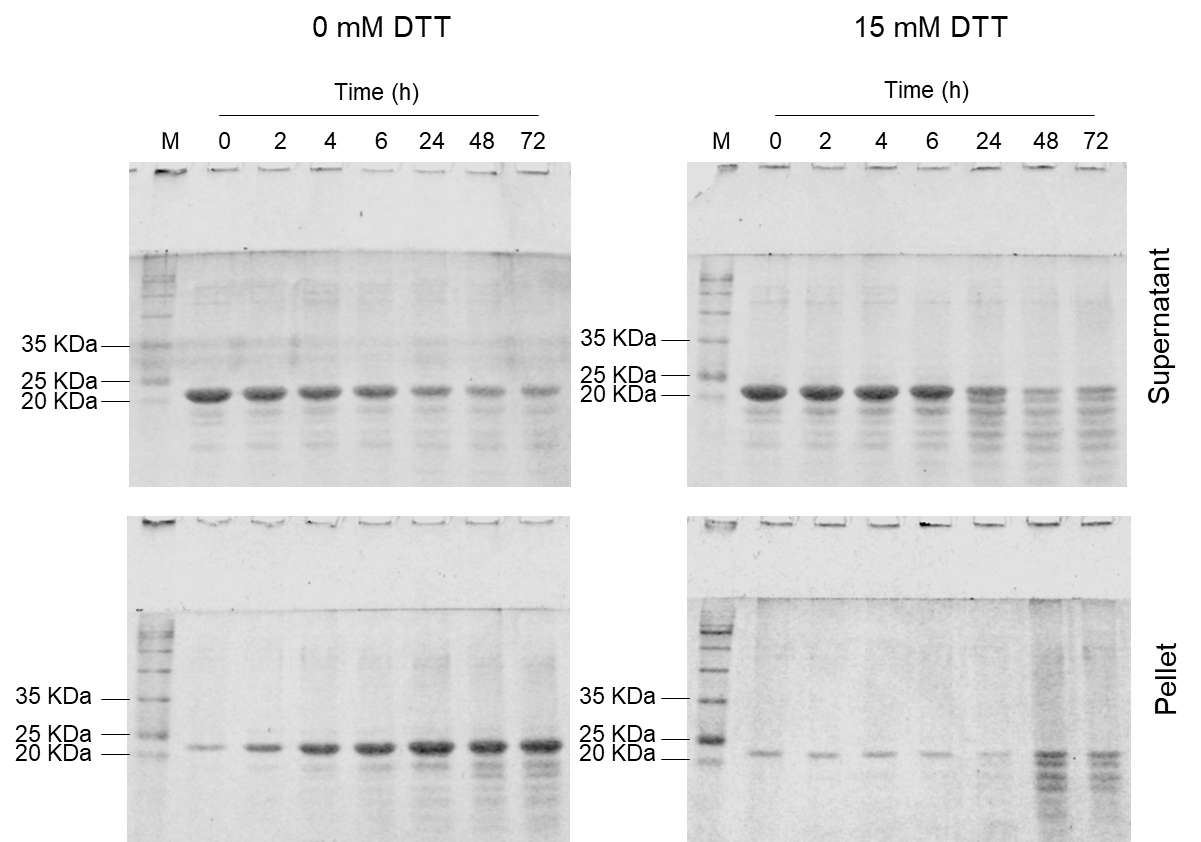
**

**Figure S1: HspB8 solubility assay under reducing conditions.** 20 µM of freshly purified HspB8 was incubated at 37°C in PBS in the presence of protease inhibitors with or without DTT 15 mM. At different times (0, 2, 4, 6, 24, 48 and 72 hours), aliquots of 20 µl were centrifuged and both supernatants and pellets were subjected to SDS-PAGE (14%). Gels were stained with EZBlue gel staining solution and scanned at 700 nm with Odyssey Fc System (LICOR).
